# The Importance, Challenges, and Possible Solutions for Sharing Proteomics Data While Safeguarding Individuals’ Privacy

**DOI:** 10.1016/j.mcpro.2024.100731

**Published:** 2024-02-07

**Authors:** Mahasish Shome, Tim M.G. MacKenzie, Smitha R. Subbareddy, Michael P. Snyder

**Affiliations:** Department of Genetics, Stanford University, Palo Alto, California, USA

**Keywords:** data sharing, repository, personal identifiers, data privacy, proteomics techniques

## Abstract

Proteomics data sharing has profound benefits at the individual level as well as at the community level. While data sharing has increased over the years, mostly due to journal and funding agency requirements, the reluctance of researchers with regard to data sharing is evident as many shares only the bare minimum dataset required to publish an article. In many cases, proper metadata is missing, essentially making the dataset useless. This behavior can be explained by a lack of incentives, insufficient awareness, or a lack of clarity surrounding ethical issues. Through adequate training at research institutes, researchers can realize the benefits associated with data sharing and can accelerate the norm of data sharing for the field of proteomics, as has been the standard in genomics for decades. In this article, we have put together various repository options available for proteomics data. We have also added pros and cons of those repositories to facilitate researchers in selecting the repository most suitable for their data submission. It is also important to note that a few types of proteomics data have the potential to re-identify an individual in certain scenarios. In such cases, extra caution should be taken to remove any personal identifiers before sharing on public repositories. Data sets that will be useless without personal identifiers need to be shared in a controlled access repository so that only authorized researchers can access the data and personal identifiers are kept safe.

## Highlights

• Proteomics data sharing is increasing but there is still large room for improvement.
• Participation from institutes, repositories, journals & funding agencies is needed.
• Awareness, incentivizing researchers, and regulations can increase data sharing.
• Removing all personal identifiers from proteomics dataset is crucial before sharing.

## Graphical Abstract

**Figure:**
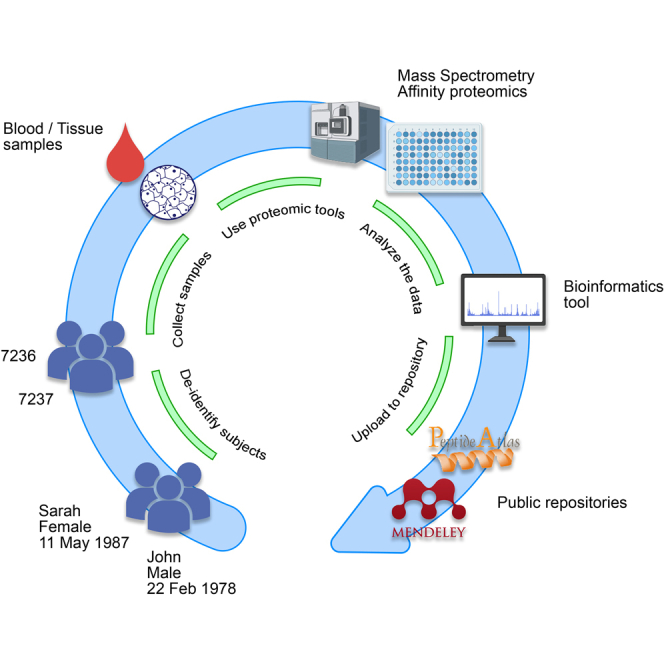

## In Brief

Proteomics data sharing is improving gradually, however there is a need for involvement of all key players to make data sharing a norm. Currently, researchers only share the bare minimum dataset required to publish their article, often without proper metadata or only partial datasets. With increased awareness, incentives, and regulations, researchers are more likely to submit data in publicly accessible repositories. Removing personal identifiers of study participants before data sharing in order to safeguard their right to privacy is important.

Data sharing in biomedical research helps accelerate discovery, increase reproducibility, and reduce research costs. The Human Genome Project (HGP) is an example where international collaboration led to the mapping of the entire human genome. This wouldn’t have been feasible if performed by a single researcher. Recently, the vaccine development of SARS-CoV-2 at an unprecedented pace was only possible due to international collaboration. The success of HGP led to the mandate of genomic data-sharing policy by the National Institutes of Health (NIH), according to which researchers are required to share large-scale human or non-human genomic data in a public repository regardless of funding level. HGP laid out the principles and was later adopted by funding agencies and journals to make data sharing a requirement in the genomics field. Unfortunately, no such mandates are in place for proteomics data. It is high time that funding agencies and journals recognize the importance of proteomics in understanding health and disease and mandate data sharing.

Proteomics can be broadly categorized into expression, functional, and structural (1). Expression proteomics is concerned with understanding differences in protein expression between different cell or tissue types, developmental time points, organisms, or health and disease states (2, 3, 4, 5). Functional proteomics is concerned with understanding the role a protein or protein complex plays within cell and organismal biology, often by identifying subcellular localization, protein–protein interactions, biological pathways, or biomarkers for disease (6, 7, 8, 9). Both expression and functional proteomics are well served by the Human Protein Atlas, which is a project aimed at mapping the entire human proteome in health and disease states (10). Structural proteomics is concerned with understanding the three-dimensional shape a protein folds into based on its amino acid sequence. Structure encodes function and can therefore provide insights into mechanistic function, ligand binding, and complex assembly.

Expression and functional proteomics are mostly studied using Mass spectroscopy (MS) or affinity-based techniques while structural proteomics is mostly studied using X-ray crystallography, cryo-electron microscopy (cryoEM), or Nuclear Magnetic Resonance (NMR). These techniques generate huge amounts of data which are mostly stored in local computers, leading to the risk of losing data and making it inaccessible to other researchers. Recent efforts from funding agencies and journals have increased the rate of proteomics data sharing. The inclusion of data availability statement and the option of data submission to online repositories by journals has had a positive impact. However, it has been found that most of the researchers do not share article data in a usable format when requested. In a study, the authors analyzed 1792 articles published in BioMed Central (BMC) journals. Upon requesting data, only 6.8% of authors handed over the data in a usable format (11, 12). In another study, authors analyzed the accessibility of data in different disciplines and compared them over 2 decades (2000–2009 and 2010–2019). Low data sharing is a widespread issue across disciplines, however, most of the disciplines saw a significant increase in full data availability from 2000 to 2009 to 2010 to 2019 (13). This observation is most likely due to the increase in journals requiring submission of data in a public repository, as well as an increase in general awareness.

Although data sharing has multiple benefits, there are some privacy risks associated with it as well. We know that personal identifiers like name, address, or phone numbers of the participants can be used by miscreants and bad-faith actors to cause harm. Therefore, such obvious identifiers must be removed before sharing the dataset in public repositories. In the US, the Health Insurance Portability and Accountability Act (HIPAA) provides guidelines to handle Protected Health Information so that an individual’s privacy is protected. In Europe, the General Data Protection Regulation provides guidelines on the handling of personal data by companies. Similar regulations exist in China and Brazil (14, 15). In other countries, Institutional Review Boards or other forms of national regulation protect PHI. In 2013, HIPAA amended its list to include genetic information as one of the PHI (16). While proteomics data is not considered PHI, expression level of individual-specific proteins, single amino acid variation (SAAV), and post-translational modification (PTM) profiles can be used to re-identify an individual in certain scenarios (17). It has been found that there are some plasma proteins whose expression level has less variation in the same individual over time compared to different individuals among themselves (18). There are other plasma proteins whose expression level is elevated in pregnant women. The expression level of these proteins can be used to re-identify individuals as well as their sex.

Despite the journal's requirement for data sharing, researchers often share data for a few sections and ignore other sections which are crucial for the full conclusion of the article. Also, the metadata is often incomplete, making the dataset unusable (19, 20). In this article, we have identified reasons for reluctance to share complete datasets, the benefits of data sharing, the importance of safeguarding participants’ privacy, and possible solutions for the mentioned challenges. We have also provided a list of various repositories available for proteomics data and their associated pros and cons in Table 1.

**Table 1:** Pros and cons of different proteomics specific repositories

Sl no.	Name	Pros	Cons
1	PRoteomics IDEntifications (PRIDE) database	▪ It is the most widely used mass spectrometry-based proteomics repository.	
		▪ Each entry has sample processing and data processing protocol, critical for reproducibility.	
2	MassIVE	▪ It encourages and facilitates re-analysis of dataset from another researcher using in-built tools.	
		▪ It has an extension for MS quantitative data storage and analysis workflow.	
3	Panorama	▪ It is integrated with Skyline workflow. Analysis results can be directly uploaded from the Skyline application. Raw data can be uploaded using PanoramaWeb.	▪ It is customized for Skyline and therefore cannot accept processed data from other software.
4	PeptideAtlas	▪ It can store tandem mass spectrometry raw data (.wiff, .raw, mzML) and metadata.	▪ Dataset cannot be directly submitted. First, it needs to be submitted to ProteomeXchange and then it can be reprocessed.
		▪ It has in-built pipeline to process spectra files and identify peptides.	▪ Software is not intuitive or user friendly.
5	Japan ProteOme STandard Repository (jPOSTrepo)	▪ It accepts both raw and processed MS data.	▪ It is a basic repository lacking additional analysis tools.
		▪ It doesn’t require additional software download. Web application has very fast data upload capabilities.	
6	iProX	▪ It contains a repository and a proteome database. It can store MS raw data and metadata.	▪ Universal Spectrum Identifier feature of the web application doesn’t work.
		▪ It is integrated with Aspera which makes the file uploading and downloading much faster.	▪ The password management system is not encrypted as the account passwords are stored as normal text in the database.
7	Proteomics Data Commons (PDC)	▪ It is developed by National Cancer Institute to make cancer-related proteomics data available to the public.	▪ It is specific for cancer and therefore cannot accept studies which are not related to cancer.
		▪ It organizes the entries based on primary site of the disease and cancer type.	
8	ProteomicsDB	▪ It assembles data from ProteomeXchange and also allows user to upload quantitative proteomics data.	▪ There are too many features and tools integrated which make it difficult to understand the main goal of the repository.
		▪ Searching for individual proteins can reveal protein coverage by MS data, interaction map, expression in human tissues, etc.	
9	Protein Microarray Database (PMD)	▪ It is dedicated for proteomics data generated by protein microarrays.	▪ It is lacking metadata regarding the sample demographics, number of subarrays and detection substrate.
		▪ It has in-built tools to compare experiment group *versus* control group data.	
10	ArrayExpress	▪ It accepts functional genomics and proteomics data generated by various types of microarray.	▪ It is not customized for proteomics data.
		▪ It has sections describing sample collection, nucleic acid extraction and normalization protocols for better reproducibility.	
11	ImmPort	▪ It accepts various types of immunological data generated by ELISA, Flow cytometry, Luminex, RNA seq etc.	
		▪ Each entry is well organized with design, assays, demographics, and raw files.	
13	Protein Data Bank (PDB)	▪ It is the biggest repository for structural proteomics. Data obtained through X-ray crystallography, NMR and electron microscopy are stored.	
		▪ It has in-built tools to convert structure factor format and also 3D visualization of proteins.	
14	Integrated Resource for Reproducibility in Macromolecular Crystallography (IRRMC)	▪ It contains the raw data from diffraction experiments that can be used to generate structures for PDB submission.	▪ Three-dimensional structure viewer for proteins is not available on the website.
		▪ X-ray diffraction datasets can be uploaded and linked to associated PDB identifier.	
15	Coherent X-ray Imaging Data Bank (CXIDB)	▪ It accepts entries where protein structures were determined using coherent X-ray beams.	▪ It lacks data uploading form and requires its user to email the data in a recommended format.
		▪ It contains instructions to change other file formats to.cxi, for standardization.	
16	Electron Microscopy Data Bank (EMDB)	▪ It accepts volume maps and tomograms of macromolecular complex using the OneDep deposition system.	▪ The website isn’t organized properly.
		▪ Information such as resolution of the structure as well as complex, protein and ligand information are included.	
17	Biological Magnetic Resonance Data Bank (BMRB)	▪ It can store spectral and quantitative data derived from NMR spectroscopy experiments.	▪ The website isn’t organized properly and isn’t intuitive.
		▪ It has detailed metadata and details about different chemical shifts.	

## Reasons for Reluctance to Share Proteomics Dataset

The rate of proteomics data sharing has increased over the years, mostly due to journal and funding agency requirements. Despite the requirement, researchers have been reluctant to share proteomics data in a usable format. Most high-impact research articles include four to five different techniques for data collection. However, the shared dataset contains just one technique dataset, and the remaining datasets are often mentioned as “available upon request from corresponding author”. In addition, the metadata is often incomplete, and the “Methods” section is so brief that important information required to re-use or reproduce the results is lost. This trend demonstrates a reluctance from researchers to share their data. The prime reasons are lack of incentives, lack of ethics, lack of awareness, and fear of losing financial gains or credits. The time and money required to relabel, reformat, and add metadata before sharing on repositories is not rewarded properly (21). Currently, data sharing doesn’t play any role in the professional development of the researcher. There are no incentives awarded for properly sharing the data. Regarding ethics, there is a moral obligation of the researcher to share the data in a way that can be re-used and re-analyzed (22). Researchers with low ethics consider sharing data in a usable format makes their findings and potential scientific misconduct vulnerable for extra scrutiny. An in-depth analysis in 2012 of thousands of retracted biomedical research articles found that scientific misconduct accounted for the majority of retractions (23, 24). The number of retracted papers has recently increased year over year as efforts are taken to root out scientific misconduct (25). Despite the fact that there are established norms with regard to research integrity and serious sanctions are in place for violation, a survey of NSF fellows indicates that early career researchers feel ill-equipped to handle ethical quandaries in their research groups (26, 27, 28, 29). This data indicates the need for better training. Even if researchers want to share all datasets, they are not aware of all the resources available to them and how these resources compare among themselves. Few opportunities to take courses and receive training related to data sharing at research institutions lead to low awareness among researchers. There is also a fear that data sharing can lead to losing financial gains and credits associated with the raw data. The shared raw data can be used to build commercial products without proper remuneration or to publish a paper without acknowledging the original data collectors. Failure to credit original authors is a clear example of plagiarism, a serious academic offense that can have major repercussions.

The datasets that are shared properly and reanalyzed by another researcher suffer from credit-sharing ambiguity. If all the researchers involved in data collection are added as co-authors in the derived article, then there will be too many authors, which will dilute the contribution of each author (30). In addition, if researchers involved in data collection are added as co-authors then they are also liable for the accuracy of the results found in the derived articles. In most cases, they don’t have any control of derived results.

## Risk of Re-Identification of Individuals From Their Proteomics Data

While data sharing is considered to be a good scientific practice, it comes with its own challenges. Sharing data recklessly can increase the amount of data shared but can compromise participants’ right to privacy. In contrast, if no data is shared for fear of re-identification, then the field of proteomics will not be able to achieve its potential impact on human health. The best way to move ahead is to adopt a balanced approach where data is shared only after safeguarding personally identifiable information (PII) (31). It is already known that genetic and transcriptomic data can be used to re-identify individuals (32). Herein, we describe the possibility of using proteomics data to re-identify individuals and its actual feasibility.

In a targeted proteomics approach, proteins which are known to be differentially expressed in individuals can be used to re-identify individuals. An abundant plasma protein vitamin-D binding protein (GC), which has three common alleles (Gc^1F^, Gc^1S^, and Gc^2^), can be used to distinguish ethnic backgrounds. Gc^1F^ is most frequently found in West Africans and African American populations and least commonly in Caucasians (33, 34). Researchers have also identified “individual-specific” proteins, whose level in plasma over time has higher intra-individual correlation compared with inter-individual correlation (35). Apolipoprotein(a) is one example of individual-specific protein which can have a 100-fold difference in expression level between individuals but remains very constant over time within individuals. Such proteins can be used to isolate or identify an individual among the set of individuals. It is also found that there is a significant elevation in estrogen-regulated proteins such as sex hormone binding globulin and pregnancy zone protein in women (35). The above findings show that plasma protein levels have the potential to identify an individual and also their sex in a small cohort. It is also important to note that there are many more proteins whose expression level in plasma changes due to disease, medication, and lifestyle. These external factors can complicate the practical feasibility of identifying specific individuals from proteomics data.

Genomic variation due to non-synonymous single nucleotide polymorphism can lead to the modification of a single amino acid in a protein sequence. Mass spectrometry-based proteomics can identify the variant peptides with SAAV. The combination of variant peptides in different proteins can be used to uniquely identify an individual. In a longitudinal study, 53 variant peptides were used to distinguish 42 individuals across different time points. It was found that 89% of all comparisons were correct samples (35). This observation highlights the increased importance of taking care to protect PII in longitudinal studies. SAAV can also be used to differentiate individuals based on their hair proteome. The allele pattern of 89 variant peptides derived from keratins, and other hair proteins were used to distinguish 66 individuals (36, 37). It was found that each individual had a very distinctive signature and very little likelihood of a random match. It is also important to note that the sample size of the above studies was limited; increasing the sample size may make it more difficult to pinpoint the individual. In the above examples of SAAV, the uniqueness of the variant peptide plays a huge role. For instance, if the variant peptide is very common then it is very difficult to identify an individual. On the other hand, if the variant peptide is very rare, then it can dramatically reduce the number of individuals with that variant peptide among the pool of individuals and therefore it will be easy to identify an individual (31).

The PTM profile of an individual can be traced back to their genomic variation. Glycosylation or phosphorylation patterns of a protein can be very different based on the gene encoding the enzyme for glycosylation or phosphorylation. Research has shown that the PTM profile of a person is distinct from others and can be used to re-identify an individual. In one study, the authors identified 95 PTMs on multiple isoforms of Tau protein in a study to identify Alzheimer's disease stages (38). It was found that the PTM profile showed heterogeneity among the individuals. It is also true that PTM can vary depending on cell type as different cell types have different sets of enzymes for PTM, therefore the feasibility of re-identification from PTM data is very low. The above examples on differential expression, SAAV, and PTM profile demonstrate that specific types of proteomics data have the potential to re-identify an individual in specific scenarios. Thus, the obvious personal identifiers like name, address, phone number, and so on must be removed. Furthermore, specific types of proteomics data should be shared with extra caution so that re-identification of participants is not possible.

## Benefits of Sharing Proteomics Data

Proteomics data contains a huge richness of information that has not been fully leveraged to reach its full potential of impacting human health (39). Data shared safely through online repositories can make it accessible for others to re-analyze and generate additional conclusions. Some of the benefits of data sharing are: improvement of science, creation of megadata, personal satisfaction through increased citations and recognition of one’s work, and opportunities for collaboration. Results that can be reproduced from raw data by other researchers will get more exposure and acceptance. Other researchers can use the results with more confidence, thereby increasing the citation of the article. According to a 2020 study on more than 50,000 articles published in *PLOS* and *BMC* journals, it has been found that articles that link their raw data *via* repositories have 25% more citations on average than those that did not (40). The increased rate of citations from articles fully sharing raw data indicates the utility of such openness to the broader scientific community. Data sharing also improves science as the original researchers are more likely to do better science because of the possibility of someone cross-checking their findings. They are also likely to carefully interpret the data and not perform a casual interpretation (41). This increases the trustworthiness of the research. For instance, Moderna Inc shared the design, efficacy, and sequence of mRNA vaccine for SARS-CoV-2, which led to more trust in the vaccine (42). Data sharing can also lead to the creation of megadata repositories, where multiple researchers working in the same field contribute. Small datasets often don’t have meaningful conclusions, but when many datasets are put together, it can reveal important conclusions. For instance, there are a lot of microbes in the human gut and very little is known about their interactions with our immune system. Looking into individual microbes and their interaction with the immune system can reveal biological pathways without any clear relation to homeostasis or disease. However, when data from different microbes are collectively analyzed, it can show the entire picture of microbial-immune interaction. This is only possible when researchers around the world share their data. Data sharing can also lead to new collaborations in the same discipline or another discipline altogether. A pathologist who collected images of cancerous tissue can collaborate with a software developer to build a predictive model to analyze those images. In such a scenario, skills from both the pathologist and the software developer are important for the success of the project.

In addition to the above benefits, data sharing can also reduce research expenditures by allowing researchers to build upon shared data without repeating the original findings. This is beneficial for both sides as preliminary researchers get more citations and researchers using the shared data reduce the research costs. In our times, high-throughput techniques generate terabytes of data, which are in the possession of few people. These datasets, if made Findable, Accessible, Interoperable, and Reusable (FAIR), then it can be accessed by researchers from various geographical locations for additional analysis (43). This process will democratize proteomics data. It is also important to note that certain datasets become useless without personal identifiers like genomic information or geographical location. In such a case, “controlled access” data sharing approach should be utilized. The idea of controlled access data sharing is currently being used to share DNA and RNA sequences of potentially sensitive data, such as germline genomic sequences (44). In this approach, a researcher can request access to potentially sensitive data from the repository. A data access committee decides the credibility of the request, identity of the researcher, and conveys the guidelines for the usage of the sensitive data (31). The researcher is prohibited from re-sharing the data and is often required to delete the data after a set period of time. This way proteomics data with PHI or PII can be used for legitimate purposes with reduced risk of potential harm to the participants.

## Repositories for Various Datatype

### Expression and Functional Proteomics

For MS data, there are multiple repositories where data can be submitted and shared. Based on the researcher location, it is best to upload the dataset to a geographically close repository as large dataset (>100 GB) transfer to a far-off location can take a long time. In Europe, PRIDE (PRoteomics IDEntifications database) is the repository that can store MS raw data, peak list files, and protein identifications data (45). It is the largest repository for MS with more than 25,000 public datasets and receives around 500 new datasets monthly. In the US, there are three main repositories for MS data: MassIVE, Panorama, and PeptideAtlas. MassIVE (Mass Spectrometry Interactive Virtual Environment) is the largest US-based repository that can store both targeted and untargeted MS data. It has built-in tools to reanalyze and link the new analysis with the original dataset (46). It also has an extension for storage and annotation of quantitative MS data. Panorama on the other hand is a repository for targeted MS data and can store the entire data model analyzed through Skyline application (47). The skyline application is used to process and analyze MS datasets, which then can be directly uploaded to the repository from the application. PeptideAtlas is a repository that can store peptides and protein information identified from tandem MS experiments (48). Deposited raw files are searched in protein databases and processed through a pipeline to determine the probability of correct identification. The spectrum of the peptide as well as the number of experiments that identified the peptide is listed in the web application. In Asia, there are two main repositories namely jPOSTrepo (Japan ProteOme STandard Repository) and iProX. jPOSTrepo provides high-speed file uploading, and flexible file management capabilities for sharing raw or processed MS data (49). There are more than 1500 public projects in the repository. iProX was developed in China as a proteome resource center to freely share MS data (50). It is composed of a repository and a proteome database. There are more than 3000 public projects in the repository.

Due to the rapid increase of MS usage in proteomics, there was a need for standardization of data format and metadata collected. A consortium named ProteomeXchange was established so that repositories that are part of the consortium follow standard data submission and dissemination pipelines (51). Currently, all the above-mentioned repositories are members of the consortium. The data shared on each repository is accessible from ProteomeCentral (proteomecentral.proteomexchange.org). In a separate effort, Human Proteome Organization-Proteomics Standards Initiative (HUPO-PSI) published the standard for MS raw data as mzML, peptide or protein identification data, and quantification data as mzTab (52, 53). The standard data formats and data submission guidelines are being followed in each repository, which has made MS-based proteomics more accessible and useful for the scientific community.

There are different affinity-based techniques that are gaining popularity in the field of proteomics. Protein microarrays, Luminex, Olink, and SomaLogic are some examples. Protein microarray data can be shared using the Protein Microarray Database (PMD), which is a dedicated application for archiving, sharing, and analyzing the data (54). As there was no dedicated repository for protein microarrays before PMD, the majority of the data was not publicly accessible. PMD encourages users to submit their raw data in GPR format (GenePix). They have in-built tools in the web application to identify differentially expressed proteins, perform protein annotation, and pathway analysis. ArrayExpress is another repository that can store protein microarray data. However, it is customized for genomics data and is not the best choice for protein microarrays. For Luminex, Olink, and SomaLogic, there is no dedicated repository. Immunology Database and Analysis Portal (ImmPort), can be used to store and share the immunological data from the above-mentioned affinity-based techniques (55, 56). ImmPort also accepts immunological data obtained from ELISA, Flow cytometry, RNA sequencing, MS, PCR, Western blot etc.

### Structural Proteomics

Structural data of proteins acquired through X-ray crystallography can be shared using Protein Data Bank (PDB). It is the first open-access digital data resource in biology and medicine and can store three-dimensional structural data of proteins and nucleic acids obtained by X-ray crystallography, cryoEM, and NMR (57). There are more than 200,000 depositions to date to the data bank, of which around 76% are protein structures obtained by X-ray crystallography. The accepted file format is mmCIF for PDB and there are tools available on their website to convert structure factor files to mmCIF format (58). The large database of high-quality experimental protein structures has been a source of training data for machine learning algorithms designed to predict protein structure directly from amino acid sequences (59). AlphaFold has created its own database containing predicted protein structures, increasing the access to structural proteomics beyond current experimental data (60).

PDB doesn’t store the raw data of protein crystallography experiments; therefore, IRRMC (Integrated Resources for Reproducibility in Macromolecular Crystallography) was created. The goal of IRRMC is to store raw data of protein crystallography experiments so that results can be reproduced (61, 62). The metadata is saved related to the dataset so that reprocessing of the diffraction data is possible. The repository contains all the crystallography binary files (CBF) that can be used to identify other lattices or strong diffuse scattering that were not analyzed by the original study. The CBF can be used to generate “.hkl” file for PDB deposition. Structural analysis of proteins that are difficult to crystallize are not possible with standard X-ray crystallography and needs coherent X-ray diffraction imaging (63). CXIDB (Coherent X-ray Imaging Data Bank) is a data bank to store coherent X-ray imaging data in CXI file format. There are more than 181 datasets in the data bank and new ones can be uploaded *via* FTP connection to the server.

CryoEM is increasingly used for high-resolution imaging of protein and protein complexes. CryoEM is proving especially useful for dynamic transmembrane proteins that have been resistant to crystallization efforts (64). Volume maps and tomograms obtained from cryoEM can be deposited to Electron Microscopy Data Bank (EMDB). The data bank is a member of wwPDB (worldwide PDB) organization and uses its deposition system. The deposition system, OneDep, is used by all member repositories of wwPDB. There are more than 21,000 protein structures available on EMDB determined through cryoEM. When the size of the protein is low (<30 kDa), the structure can also be determined using NMR. NMR structural data can be quite effective at capturing protein dynamics and ligand-induced conformational shifts (65, 66). The data obtained from NMR instruments can be stored in Biological Magnetic Resonance Data Bank (BMRB). The data bank annotates and archives NMR spectral and quantitative data derived from peptide, protein, and other biological molecules (67). NMR spectral data with and without coordinates can be deposited through wwPDB’s OneDep system and BMRB’s inherent deposition system. There are more than 7300 entries of protein structures determined using NMR in the repository.

### General Purpose Repository

General purpose repositories are file storage systems that lack customized tools or viewers found in technique-specific repositories. Some of the most common ones are Figshare, Zenodo, Open Science Framework (OSF), Harvard Dataverse, Mendeley Data, PlutoF, and Dryad. They accept various file formats and are mostly free to use. Figshare provides 20 GB free storage space, and an extra five TB can be obtained by paying a fee. All the data that is uploaded and published gets a digital object identifier (DOI) (68). Zenodo is hosted in CERN’s (European Organization of Nuclear Research) data center, and it provides free storage up to 50 GB per dataset. If there is a requirement for additional storage space then it can be requested by contacting them (69). OSF is a project management tool that can be used to organize, share, and store data for the long-term (70). Projects can be made public and a DOI can be requested for reference. PlutoF is designed for storing and managing biodiversity data ranging from taxonomy, ecology, phylogeny, and so on. (71). It contains a file repository through which datasets can be published and a DOI can be requested. Dryad can store multiple file formats and can publish those datasets for a publishing charge of $150 (72). If the dataset size exceeds 50 GB, then it will incur additional charges. Member research institutes, low-income, and lower-middle-income economies are exempt from data publishing charges.

## Discussion

**Fig. 1:**
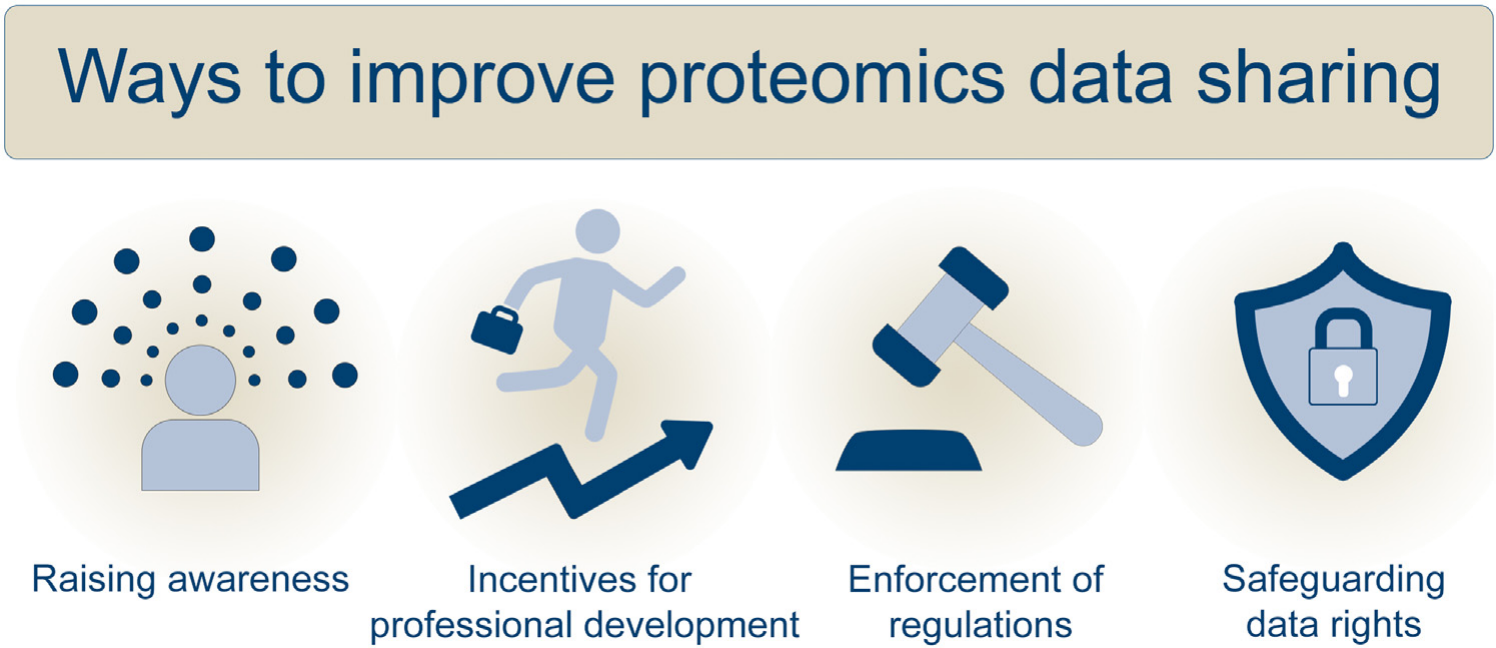
**Different approachesthat can help improve proteomics data sharing.**

Sharing of proteomics data is crucial for the advancement of the field and its broader benefits to the scientific community. The rate of data sharing has steadily increased over the past decade, mostly due to journal and funding agency requirements. Journals that do not require data to be shared in a repository often have “data availability statement” where the authors promise to share their data upon request. When requested, they mostly don’t share. Journals that require data to be shared in a public repository are better in the reproducibility of results. However, it has been found that authors just provide the bare minimum data required for article publishing. Often, they don’t share all the datasets used in various techniques or metadata is incomplete. First, there is a need for incentivizing researchers who share the data in a usable format (Fig. 1). They can be incentivized for proper data sharing by awarding points, which will be helpful in their professional development (tenure or promotion review). One of the principles in the widely recognized San Francisco Declaration on Research Assessment calls for the recognition of scientific outputs beyond publications, explicitly highlighting the positive value of sharing datasets (73). Second, it is also the responsibility of research institutes to educate the researchers about using repositories suitable for their techniques, available resources, and their benefits. When researchers realize the benefits of data sharing both for themselves and the scientific community, they are more likely to do it willingly. Thirdly, regarding the fear of losing credits, researchers using the data for re-analysis need to properly cite the original dataset. If found otherwise, the article can be retracted. Plagiarism is a serious form of academic misconduct that can have further career impacts beyond the retraction of an article. If a company plans to use the original dataset for financial gains, then it needs to gather appropriate rights from the original data collector beforehand. If found otherwise, legal action can be taken against the company to share the profit or pay a fine. Lastly, peer-reviewers and editorial team of the journals need to verify that datasets for all techniques are uploaded and proper metadata is provided before publishing the article.

New regulations for data sharing are getting mandated by funding agencies and journals. Since 2003, NIH has required researchers to share their data in a public repository if the direct cost of the grant is over $500,000 (21). From 2023, NIH policy mandates data-sharing plans for all NIH sponsored grants irrespective of grant amount. These data-sharing plans will inculcate awareness of the importance and mechanisms for data sharing for early career researchers applying for F and K fellowships. Journals like *Science* and *PLoS* have mandatory data-sharing policies. They require authors to share data necessary to replicate the findings of the article before publication. *Science* has partnered with Dryad to cover the cost of hosting the data of accepted articles in Dryad repository. *PLoS* doesn’t cover the repository fee but uploads the supporting information data of accepted articles to Figshare repository to increase FAIR compliance. More and more journals are following the trend and making data sharing mandatory.

Safeguarding the privacy of individuals participating in the research study is the responsibility of all researchers who are part of the project. Any personal identifiers that alone or in conjugation with other identifying information can re-identify an individual must be removed. Expression levels of individual-specific proteins, SAAVs, PTM profiles should be handled with extra care as they have the potential to re-identify an individual in certain scenarios. Several repositories also ask if the obvious personal identifiers are removed before submission. In rare cases when removing personal identifiers can make the dataset useless, then repositories with controlled access capabilities should be used. Researchers with genuine credentials can request access to the data and analyze the data, but they are not allowed to re-distribute it. This way the complete dataset is shared as well as sensitive information is kept safe.

For MS data, there are more options to store and share the data compared to other techniques. Due to ProteomeXchange consortium, the repositories which are members of it have proper data dissemination pipelines. They also follow similar data formats and metadata. In contrast, emerging techniques such as protein microarrays, Luminex, Olink and SomaLogic don’t have many repository options or standardized file formats. As these techniques gain more popularity, there will be more repository options and standardization in the future. Apart from the repositories that are mentioned in this article, we found that some of the repositories were not updated in a long time, or their website is no longer accessible. This is mostly the case when the repository is funded and maintained by a single research group. Due to the lack of resources, prolonged maintenance of the repository is not feasible and therefore is shut down. Repositories that are started by a single research group should try to find governmental or organizational funding for prolonged survival. It is also crucial that these repositories use data formats that are widely accepted so that in case of repository closure, the data can be easily transferred to another repository.

For general-purpose repositories, we found that some of them have a fee for data storage. While it is understandable that maintaining such data banks requires money but asking the depositor to pay a hefty fee can discourage them from submitting the data. A subscription package that is applicable to an entire institution or department is a more viable option. These repositories should also ask for mandatory README files for each project. This way anyone using the dataset can understand the organization of folders and the significance of all files in the project. In conclusion, we strongly believe that data shared after safeguarding the participants’ privacy is crucial for scientific advancement. There is still a lot of room for improvement, which can be addressed collectively by research institutions, funding agencies, journals, repositories, and researchers themselves.

## Data Availability

No data or funding was used for this article.

## Conflict of interest

The authors declare that they have no known competing financial interests or personal relationships that could have appeared to influence the work reported in this paper.
